# Supplementary figures and images for: Phosphorylation of PSD-95 at serine 73 in dCA1 is required for extinction of contextual fear
Source: PLoS Biol. 2023 May 8;21(5):e3002106. doi: 10.1371/journal.pbio.3002106 (PMC10194913; doi:10.1371/journal.pbio.3002106)

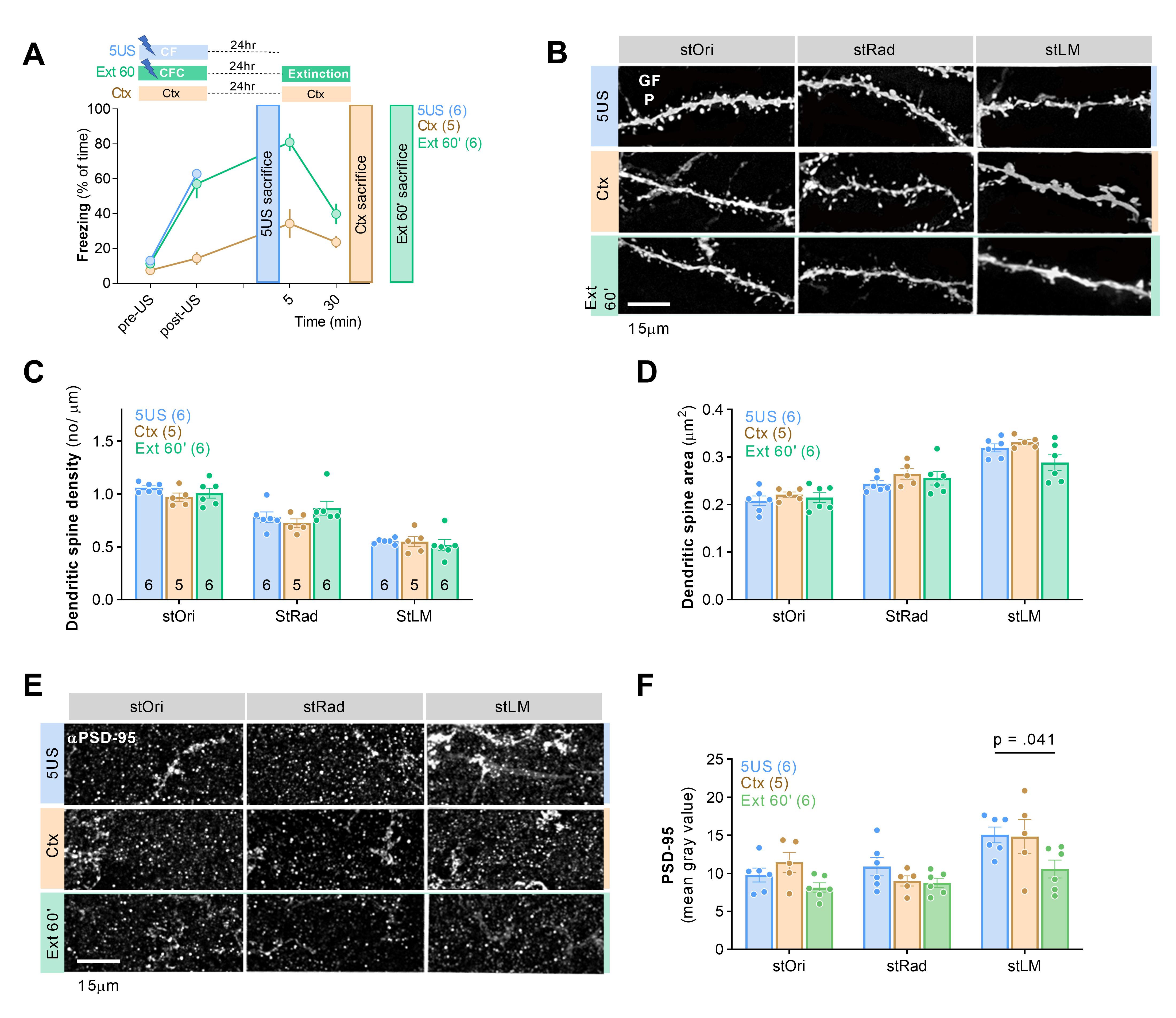

Supplement: S1 Fig — Dendritic spines were analysed in 3 domains of dendritic tree of dCA1 area in Thy1-GFP(M) male mice: stOri, stRad, and stLM. (A) Experimental timeline and freezing levels of mice from 3 experimental groups: 5US (mice killed 1 day after CFC; n = 6), Ctx (mice killed immediately after the second exposure to novel context, no foot shocks were delivered, n = 5) and Ext 60’ (mice killed 60 minutes after contextual fear extinction session, n = 6). (B) Representative confocal images of dendrites (GFP) (maximum projections of z-stacks composed of 20 scans) are shown for 3 domains of the dendritic tree. (C) Summary of data showing dendritic spine density (repeated-measures ANOVA, effect of training: F(2, 14) = 1.620, P = 0.233). (D) Summary of data showing average dendritic spine area (repeated-measures ANOVA, effect of training: F(2, 14) = 3.162, P = 0.074). For C, D, each dot represents 1 mouse. (E) Representative confocal images of PSD-95 immunostaining (maximum projections of z-stacks composed of 20 scans) are shown for 3 domains of the dendritic tree. (F) Summary of data showing total PSD-95 levels (repeated-measures ANOVA with post hoc Tukey test, effect of training: F(2, 14) = 2.72, P = 0.100; effect of region: F(1.34, 18.7) = 25.1, P < 0.001; training × region interaction: F (4, 28) = 2.79, P = 0.045). For C, D, F means ± SEM are shown. The data underlying this figure are available from OSF (https://osf.io/cgfa9/). CFC, contextual fear conditioning; dCA1, dorsal CA1; PSD-95, postsynaptic density protein 95; stLM, stratum lacunosum-moleculare; stOri, stratum oriens; stRad, stratum radiatum. (TIF) [file pbio.3002106.s001.tif]

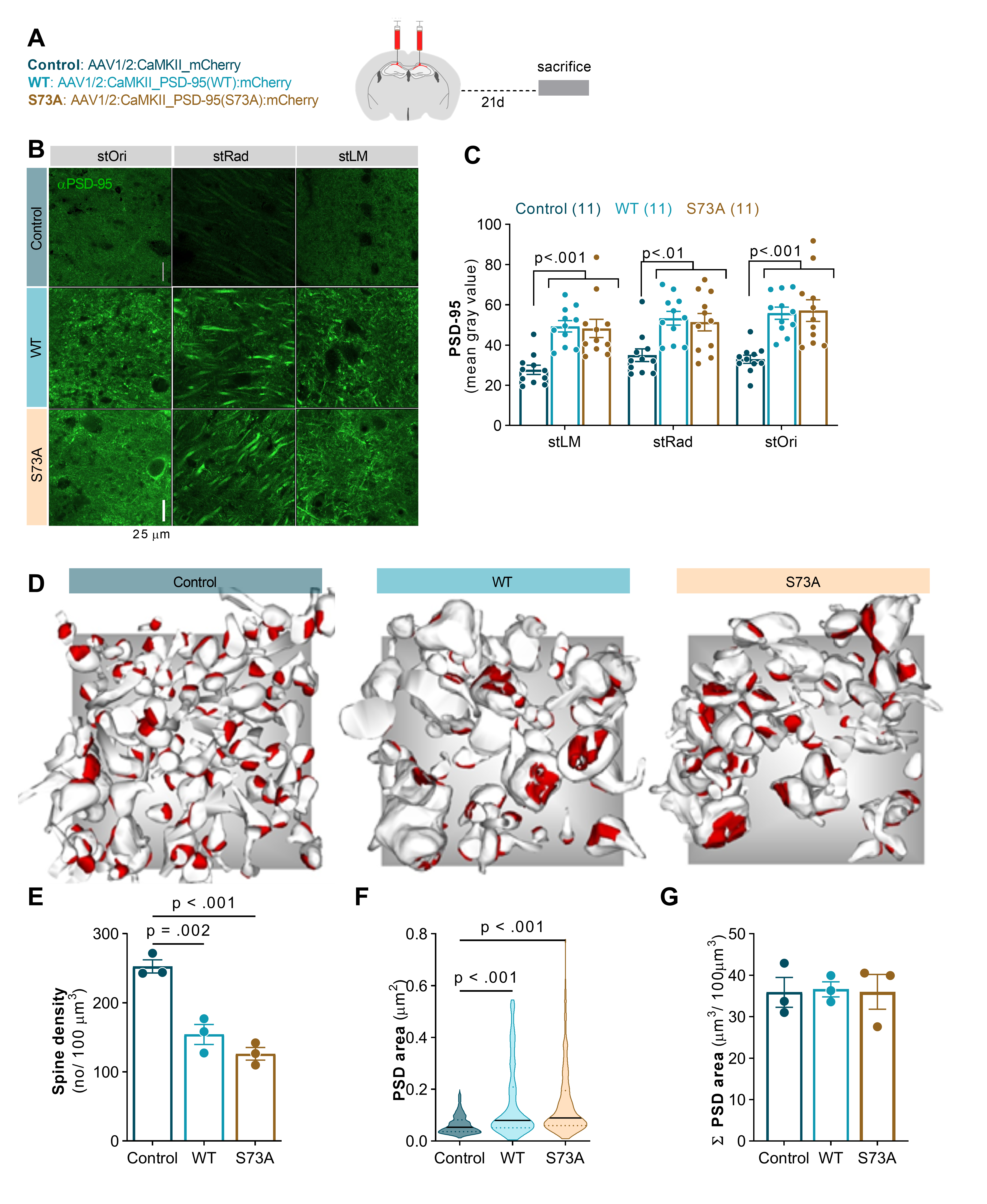

Supplement: S2 Fig — (A) Experimental timeline. C57BL/6J male mice were stereotactically injected in the dCA1 with AAV1/2 encoding mCherry (Control, n = 11) PSD-95(WT) (WT, n = 11) or PSD-95(S73A) (S73A, n = 11). Twenty-one days later, they were killed. (B) Representative confocal scans of the PSD-95 immunostaining in dCA1 strata and (C) summary of data showing PSD-95 levels (two-way ANOVA with Tukey’s post hoc test, effect of virus: F(2, 30) = 13.1, P < 0.001). (D) Exemplary reconstructions of dendritic spines and their PSDs from SBEM scans in stOri. The grey background rectangles are x = 3 × y = 3 μm. Dendritic spines and PSDs were reconstructed and analysed in tissue bricks (3 × 3 × 3 μm). (E-G) Summary of SBEM data showing: (E) mean density of dendritic spines (one-way ANOVA with post hoc Tukey test, effect of virus: F(2, 6) = 34.6, P < 0.001); (F) median PSD surface area (Kruskal–Wallis test with Dunn’s multiple comparisons test, Kruskal–Wallis statistic = 109, P < 0.001), and (G) total PSD area per tissue brick (one-way ANOVA, effect of virus: F(2, 6) = 0.0135, P = 0.9870). The data underlying this figure are available from OSF (https://osf.io/cgfa9/). dCA1, dorsal CA1; PSD, postsynaptic density; PSD-95, postsynaptic density protein 95; S73, Serine 73; SBEM, serial block-face scanning electron microscopy; stOri, stratum oriens; WT, wild-type. (TIF) [file pbio.3002106.s002.tif]

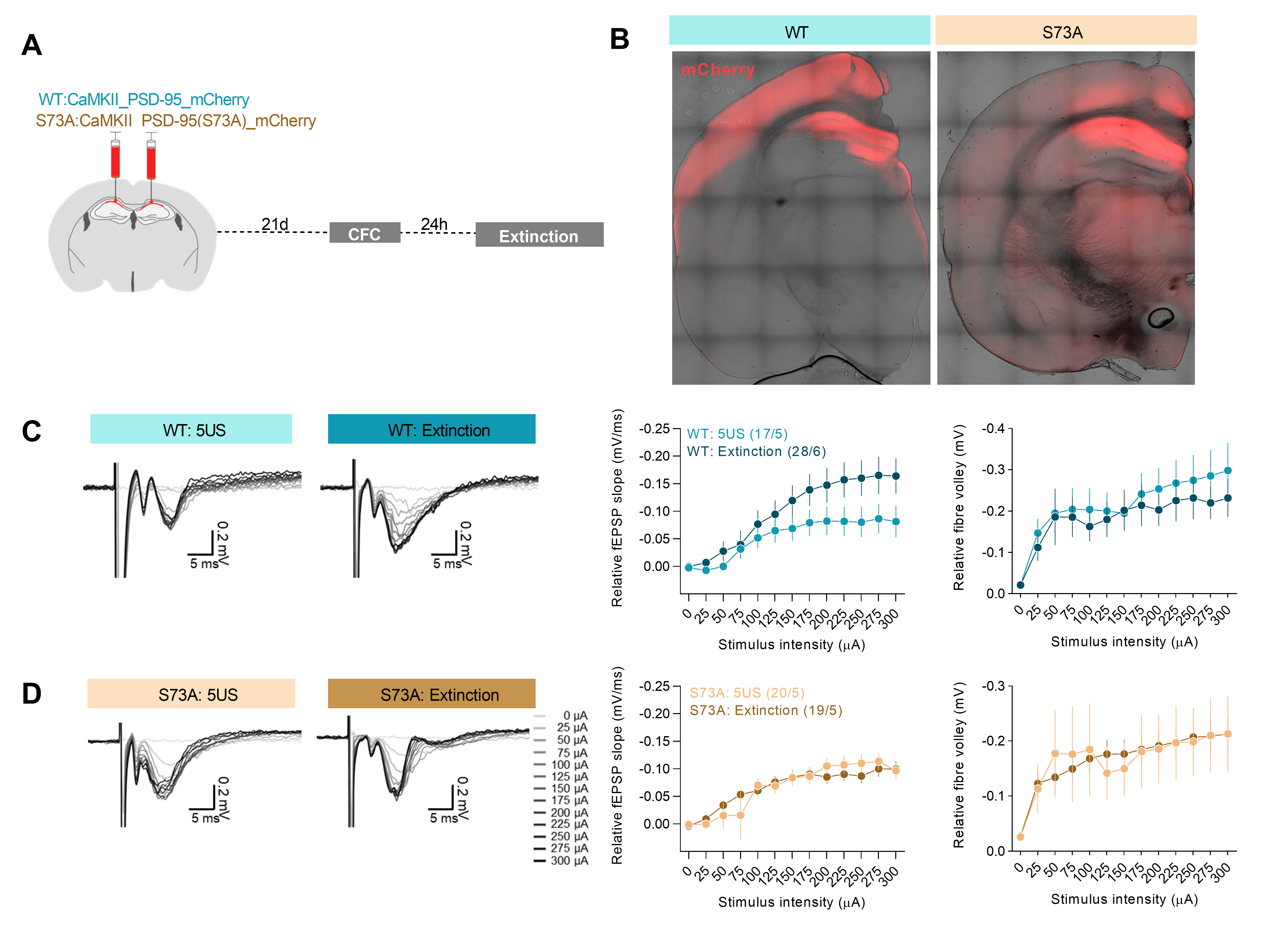

Supplement: S3 Fig — (A) Experimental timeline. C57BL/6J male mice were stereotactically injected in the dCA1 with AAV1/2 encoding PSD-95(WT) (WT, n = 11) or PSD-95(S73A) (S73A, n = 11). Twenty-one days later, they were trained and killed 24 hours after CFC or immediately after the Extinction session. (B) Microphotographs of the brain sections with AAVs expression in dCA1. (C, D) Representative fEPSPs evoked by stimuli of different intensities and summary of data in WT and S73A mice after and before fear extinction. (C) Input–output functions for stimulus intensity in WT mice (repeated-measures ANOVA, effect of virus × stimulus interaction, F(12, 456) = 2.73, P = 0.001) and fibre volley recorded in response to increasing intensities of stimulation (repeated-measures ANOVA, effect of virus × stimulus interaction, F(12, 384) = 0.467, P = 0.933). (D) Input–output functions for stimulus intensity in S73A mice (repeated-measures ANOVA, effect of virus × stimulus interaction, F(12, 456) = 1.50, P = 0.120) and fibre volley recorded in response to increasing intensities of stimulation (repeated-measures ANOVA, effect of virus × stimulus interaction, F(12, 441) = 0.412, P = 0.959). The numbers of the analysed sections/mice per experimental group are indicated in the legends. Means ± SEM are shown on the graphs. The data underlying this figure are available from OSF (https://osf.io/cgfa9/). CFC, contextual fear conditioning; dCA1, dorsal CA1; fEPSP, field excitatory postsynaptic potential; PSD-95, postsynaptic density protein 95; S73, Serine 73; WT, wild-type. (TIF) [file pbio.3002106.s003.tif]

# Raw image for Figure 3A.

WB:  $\alpha$ -phospho-PSD-95(S73)

**M**

**N**

**5US**

**Ext15'**

**x**

180kDa

130kDa

100kDa

75kDa

63kDa

48kDa

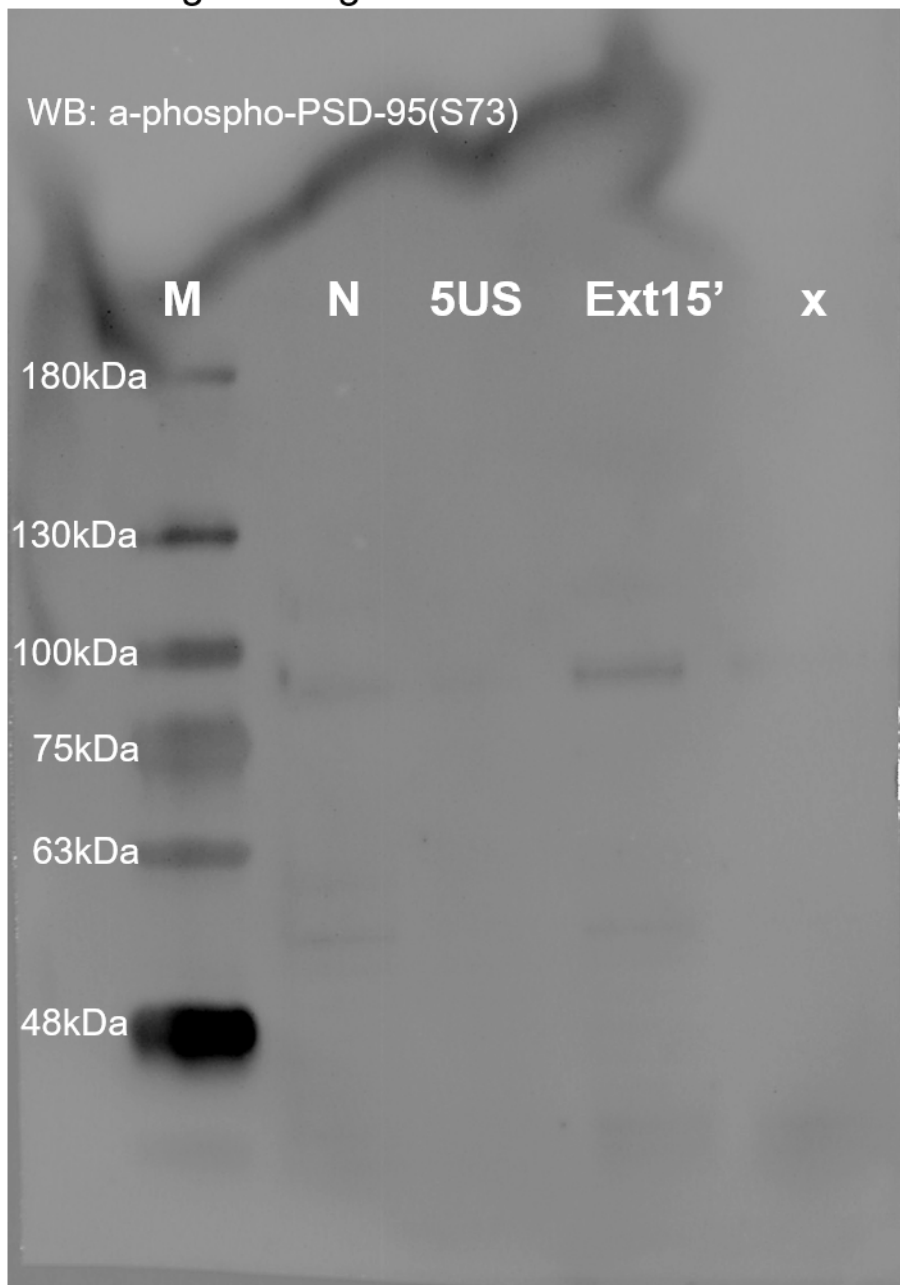

Supplement: S1 Raw Image — M, molecular weight marker; N, naive mouse; 5US, mouse that underwent CFC and was killed 24 hours later, Ext15’, mouse that underwent CFC and was killed after 15 minutes of a fear extinction session; x, sample not related to the study. (PDF) [file pbio.3002106.s004.pdf]
