# Supplementary material for: Phosphorylation of PSD-95 at serine 73 in dCA1 is required for extinction of contextual fear
Source: PLoS Biol. 2023 May 8;21(5):e3002106. doi: 10.1371/journal.pbio.3002106 (PMC10194913; doi:10.1371/journal.pbio.3002106)
Supplement: S1 Table — The table includes information about key materials used in the study. (DOCX) [file pbio.3002106.s005.docx]

| **Reagent type (species) or resource** | **Designation** | **Source or reference** | **Identifiers** | **Additional information** |
| --- | --- | --- | --- | --- |
| strain, strain background (*Mus musculus,* male) | Thy1-GFP(M) | **PMID: 11086982** |  |  |
| strain, strain background (*Mus musculus,* male) | C57BL/6J | Białystok University, Poland | IMSR_JAX:007788 |  |
| antibody | mouse PSD-95 primary antibody | Millipore  MAB1598 | RRID:AB_11212185 | 1:500 |
| antibody | rabbit P-Ser73_PSD-95 primary antibody | Davids Biotechnologie | A061 | 1:12 |
| antibody | Donkey anti-Mouse IgG alexa fluor 555 | Invitrogen A31570 | RRID:AB_2536180 | 1:500 |
| antibody | Rabbit anti-mCherry | Abcam, ab167453 | RRID:AB_2571870 | 1:500 |
| antibody | Donkey anti-rabbit Alexa Fluor 555 | Invitrogen,A-31572 | RRID:AB_162543 | 1:500 |
| antibody | Donkey anti-rabbit Alexa Fluor 647 | Invitrogen, A31573 | RRID:AB_2536183 | 1:500 |
| recombinant DNA reagent | AAV1/2, *Camk2a* _PSD-95(WT):mCherry (PSD-95(WT)) | PMID: **32029829** |  | *Dlg4* cloned into Addgene plasmid # 114469 |
| recombinant DNA reagent | AAV1/2, *Camk2a*_PSD-95(S73A):mCherry (PSD-95(S73A)) | PMID: **32029829** |  | *Dlg4*_S73A cloned into Addgene plasmid # 114469 |
| recombinant DNA reagent | AAV1/2, *Camk2a* _mCherry (mCherry) | Addgene plasmid # 114469 | RRID:Addgene_114469 |  |
| software, algorithm | ImageJ (Fiji) | PMID: 22743772 | RRID:SCR_002285 |  |
| software, algorithm | Med-PC V Software Suite | Med Associate Inc. | SOF-736 | fear conditioning software |
| software, algorithm | Reconstruct | PMID: **15817063** | RRID:SCR_002716 |  |
