# Supplementary material for: Phosphorylation of PSD-95 at serine 73 in dCA1 is required for extinction of contextual fear
Source: PLoS Biol. 2023 May 8;21(5):e3002106. doi: 10.1371/journal.pbio.3002106 (PMC10194913; doi:10.1371/journal.pbio.3002106)
Supplement: Attachment — Submitted filename: 131769_1_rebuttal_2565124_rrbtx6.docx [file pbio.3002106.s006.docx]

**Manuscript number:** #RC-2022-01751

**Corresponding author(s):** KASIA, RADWANSKA

## 1. General Statements

We would like to thank the reviewers for their valuable comments. We believe that we can provide all requested revisions.

## 2. Description of the planned revisions

*Reviewer #1 (Evidence, reproducibility and clarity (Required)):*

*The study sought to determine whether hippocampal PSD-95 is involved in extinction of contextual fear memory in mice. Although there is considerable work implicating the hippocampus in contextual fear extinction, this study adds to the literature in an important way by identifying an important role for dendritic PSD-95 in this process. The authors observe changes in PSD-95 expression and phosphorylation in dendritic spines in CA1. Disrupting phosphorylation of PSD-95 attenuated fear extinction. These are interesting data, though the lack of behavioral controls for mere context exposure renders the results difficult to interpret.*

**ANSWER**: The analysis of dendritic spines in Contextual controls (without US) vs 5US (24 hr after fear conditioning) is presented in the manuscript in Supplementary Figure 1. Unlike in the comparison Extinction vs 5US, we found no significant differences here between the groups. In the revised manuscript we will add the analysis of PSD-95 levels in the same group.

*Reviewer #1 (Significance (Required)):*

*The strengths of the report include a careful assessment of the role for hippocampal PSD-95 in contextual fear extinction, using several methods. The neurobiological assessments and interventions are robust. The primary concern is with the behavioral methodology, particularly the absence of a context-exposure control (e.g., a non-conditioned group that is treated identically to the current Ext group., or a conditioned group that is exposed to another context). This control is necessary to interpret the initial experiments, because changes in PSD-95 protein and phosphorylation may not be due to extinction learning per se, but rather to exposure to the context (and learning about that context that is independent of extinction). Thought the disruption of PSD-95 phosphorylation in the dorsal hippocampus appears to blunt context extinction with multiple extinction sessions, it did not impede the extinction procedure used in the initial experiments.*

**ANSWER**: The analysis of dendritic spines in Contextual controls (without US) vs 5US (24 hr after fear conditioning) is presented in the manuscript in Supplementary Figure 1. Unlike in the comparison Extinction vs 5US, we found no significant differences here between the groups. In the revised manuscript we will add the analysis of PSD-95 levels in the same group.

*Reviewer #2 (Evidence, reproducibility and clarity (Required)):*

*Summary:*

*In this manuscript, the authors are looking into the effects of PSD-95 phosphorylation at S73 in dorsal CA1 on extinction of fear memories. To do so, they use behavioral, immunofluorescence and electron microscopy approaches. They find that S73 phosphorylation is essential for plasticity related changes mediating fear memory extinction. In general, the data is interesting and experiments appear to have been done with good rigor. However, some experiments lack important controls. Previous literature on the effects of PSD-95 overexpression, which is central to the current study, is not discussed much. Regarding the writing, several typos were found and there are also several instances of overstatements.*

*Major points:*

*PSD-95 overexpression is documented to cause an increase in spine density and an increase in spine size (El Husseini, Science, 2000). From the data presented it is not clear that this is happening or not in your animals. The only time where animals injected with a control virus are compared with animals injected with WT PSD-95 and PSD-95 S73A is in figure 5 and no data is shown about PSD-95 amounts in these animals. Moreover, PSD-95 overexpression blocks LTP and enhances LTD (Stein J.Neurosci 2003). This would suggest that animals injected with WT PSD-95 would have deficits in memory acquisition and enhanced extinction. Please comment on this.*

**ANSWER**: The data regarding PSD-95 amounts in the Control, WT and S73A groups are shown on the Supplementary Figure 3. In the revised manuscript we will add the electron microscopy data demonstrating dendritic spine and PSD volume and density in these three groups and discuss the effects of PSD-95 overexpression on LTP and memory. The mentioned literature will be included in the discussion. However, it is important to note that predominantly *in vitro* studies exist with regards to PSD-95 overexpression. *In vivo,* although we observed a significant effect of PSD-95 overexpression on dendritic spine density and average PSD volume, the total PSD volume per tissue brick is not affected. Thus compensatory changes may explain why memory formation is not impaired in WT groups.

*In figures 1, 2, 3 and 6 PSD-95 immunofluorescence is used to quantify PSD-95 in the dorsal CA1. From these measurements, several metrics are extracted (PSD-95+ density; total PSD-95; PSD-95+, PSD-95+ puncta...) and they slightly differ in the different figures. Some of these are easy to understand but others would benefit from a more detailed description. Please use consistent metrics and/or provide a rationale for using each of the different analysis methods.*

**ANSWER**: We will clearly explain the rationale for each metrics used and explain how they were defined in the methods section.

*In Figure 6, immunostaining with the antibody specific for PSD-95 S73 needs to be done in order to link CaMKII to this story.*

**ANSWER**: The immunostaining comparing phospho-S73 levels in WT and T286A mice will be added in the revised manuscript.

*Line 220: No differences were observed in PSD-95 levels between mice with WT PSD-95 expression and PSD-95 (S73A). What about differences in PSD-95 levels in mice without viral injection, ie what is the level of overexpression?*

**ANSWER**: We apologize for this mistake in description. We did observe around 40% of overexpression of PSD-95 in WT and S73A groups as compared to the Control. This data is presented in Supplementary Figure 3. In the revised version this will be clearly stated in the results section with the reference to the Supplementary Figure 3.

*Line 270: 'The S73A mutation impaired fear extinction-induced downregulation of dendritic spine density as well as dendritic spine and PSD growth'*

*What about the effect of the S73A mutation on the same metrics (Dendritic spine volume, PSD area and PSD volume)? It looks like the 5US groups are different.*

*Also, in S73A mice, the PSD area does significantly increase, which is contrary to the statement above; please explain.*

**ANSWER**: PDS surface area is indeed larger in S37A mice after extinction, however not the volume of the dendritic spines and PSDs. This statement will be corrected to precisely state our observations.

*In the example picture shown in Figure 1 DEF, spine density and amount of PSD-95 puncta are visibly much lower in the stLM of conditioned animals (5US), this is not what is shown in the quantification at all (Figure 1GHIJ). Please provide an explanation for this and a representative example picture. Also, it would be good to show another example in a supplementary figure.*

**ANSWER**: The quantification is correct. Indeed the stLM 5US image in the current version of the manuscript looks misleading. In the revised version we will submit another picture which better represents the quantification of dendritic spines and PSD-95 in this group.

*Minor points:*

*Figure 4D: Impossible to see what is happening here; please present less (3-5) isolated examples of dendritic spines for each condition.*

**ANSWER**: We will provide isolated reconstructions of dendritic spines.

*In the introduction, it is stated at line 75 that: ' Phosphorylation of PSD-*

*95(S73) enables PSD-95 dissociation from the complex with GluN2B'. Another study found that PSD-95-S73A expression blocked the reduction in the NMDAR/PSD-95 interaction during chemical LTP in a manner that is dependent on CaMKII and calpain (Dore et al Plos One, 2014). This is consistent with the current study and the Steiner et al 2008 paper as well and should thus be mentioned and included in the citations.*

**ANSWER**: We would like to thank the reviewer for reminding us of this important study in line with the current results. We will now include it in the discussion of our results.

*Line 268: 'synaptic changes observed in the WT mice resembled the changes found in Thy1-GFP(M) animals after contextual fear extinction'. Please be more specific, sim ilarities were found in stratum oriens for the Thy1-GFP animals, which is where the SBEM experiments were done for Fig.4. What metrics exactly are similar?*

**ANSWER**: This is indeed an imprecise statement and will be corrected. We will indicate that the analysis of dendritic spines by confocal microscopy in Thy1-GFP and EM in WT mice observed decreased density of dendritic spines and increased volume of the remaining dendritic spines in stOri after extinction.

*Figure 2A: What is the signification of the H1, H2, H3 samples? Are these different mice? Why is there no band in the H2 sample?*

**ANSWER**: This information will be added.

*Line 65: PSD-95 is a major scaffolding protein at glutamatergic synapses*

**ANSWER**: This will be corrected.

*Line 106: formation of fear extinction memory => extinction of fear memory*

**ANSWER**: This will be corrected.

*Line 112: assess*

**ANSWER**: This will be corrected.

*Line 211: 30-minute*

**ANSWER**: This will be corrected.

*Line 460: co-localizes*

**ANSWER**: This will be corrected.

*Reviewer #2 (Significance (Required)):*

*This paper provides a new molecular mechanism underlying the extinction of fear memories. It should thus be of interest for the general neuroscience community, especially for people working on synaptic plasticity and fear conditioning.*

## 3. Description of the revisions that have already been incorporated in the transferred manuscript

## 4. Description of analyses that authors prefer not to carry out
