# Supplementary material for: Phosphorylation of PSD-95 at serine 73 in dCA1 is required for extinction of contextual fear
Source: PLoS Biol. 2023 May 8;21(5):e3002106. doi: 10.1371/journal.pbio.3002106 (PMC10194913; doi:10.1371/journal.pbio.3002106)
Supplement: Attachment — Submitted filename: PLOS Biol_rebuttal.docx [file pbio.3002106.s008.docx]

**Manuscript number:** #RC-2022-01751

**Corresponding author(s):** KASIA, RADWANSKA

## **1. General Statements**

We would like to thank the reviewers for their valuable comments. We believe that we can provide all requested revisions.

## **2. Description of the planned revisions**

*Reviewer #1 (Evidence, reproducibility and clarity (Required)):*

*The study sought to determine whether hippocampal PSD-95 is involved in extinction of contextual fear memory in mice. Although there is considerable work implicating the hippocampus in contextual fear extinction, this study adds to the literature in an important way by identifying an important role for dendritic PSD-95 in this process. The authors observe changes in PSD-95 expression and phosphorylation in dendritic spines in CA1. Disrupting phosphorylation of PSD-95 attenuated fear extinction. These are interesting data, though the lack of behavioral controls for mere context exposure renders the results difficult to interpret.*

**ANSWER**: The analysis of dendritic spines and PSD-95 in Contextual controls (without US) vs. 5US (24 hr after fear conditioning) is presented in the manuscript in Supplementary Figure 1. Unlike in the comparison of the Extinction vs 5US, we found no significant differences in dendritic spine metrics between the experimental groups.

*Reviewer #1 (Significance (Required)):*

*The strengths of the report include a careful assessment of the role for hippocampal PSD-95 in contextual fear extinction, using several methods. The neurobiological assessments and interventions are robust. The primary concern is with the behavioral methodology, particularly the absence of a context-exposure control (e.g., a non-conditioned group that is treated identically to the current Ext group., or a conditioned group that is exposed to another context). This control is necessary to interpret the initial experiments, because changes in PSD-95 protein and phosphorylation may not be due to extinction learning per se, but rather to exposure to the context (and learning about that context that is independent of extinction). Thought the disruption of PSD-95 phosphorylation in the dorsal hippocampus appears to blunt context extinction with multiple extinction sessions, it did not impede the extinction procedure used in the initial experiments.*

**ANSWER**: The analysis of dendritic spines and PSD-95 in Contextual controls (without US) vs. 5US (24 hr after fear conditioning) is presented in the manuscript in Supplementary Figure 1. Unlike in the comparison of the Extinction vs 5US, we found no significant differences in dendritic spine metrics between the experimental groups.

*Reviewer #2 (Evidence, reproducibility and clarity (Required)):*

*Summary:*

*In this manuscript, the authors are looking into the effects of PSD-95 phosphorylation at S73 in dorsal CA1 on extinction of fear memories. To do so, they use behavioral, immunofluorescence and electron microscopy approaches. They find that S73 phosphorylation is essential for plasticity related changes mediating fear memory extinction. In general, the data is interesting and experiments appear to have been done with good rigor. However, some experiments lack important controls. Previous literature on the effects of PSD-95 overexpression, which is central to the current study, is not discussed much. Regarding the writing, several typos were found and there are also several instances of overstatements.*

*Major points:*

*1. PSD-95 overexpression is documented to cause an increase in spine density and an increase in spine size (El Husseini, Science, 2000). From the data presented it is not clear that this is happening or not in your animals. The only time where animals injected with a control virus are compared with animals injected with WT PSD-95 and PSD-95 S73A is in figure 5 and no data is shown about PSD-95 amounts in these animals. Moreover, PSD-95 overexpression blocks LTP and enhances LTD (Stein J.Neurosci 2003). This would suggest that animals injected with WT PSD-95 would have deficits in memory acquisition and enhanced extinction. Please comment on this.*

**ANSWER**: The data regarding PSD-95 amounts in the Control, WT and S73A groups are shown on the **Supplementary Figure 2**. In the revised manuscript we also added the electron microscopy data demonstrating dendritic spine volume and density, median PSD surface area as well as total PSD surface area per tissue brick in stOri in these three groups (**Supplementary Figure 2D-G**) and in C57BL/6J mice without the virus (**Figure** **2**). We found a significant effect of PSD-95 overexpression (both WT and S73A) on dendritic spine density and average PSD area. In particular we observed decreased spine density and increased PSD area in both PSD-95 groups. The total PSD area per tissue brick is not affected. Hence, the PSD-95 effects reported by El Husseini et al. (2000) are likely specific for *in vitro* conditions. Moreover, synaptic changes observed in WT mice after fear extinction closely resembled the changes in C57 mice, indicating that training-induced plasticity was preserved after PSD-95(WT) overexpression.

In the revised manuscript, we also added an experiment investigating synaptic transmission in stOri before and after fear extinction in the S73A and WT groups (**Supplementary Figure 3**). Our data show that fEPSP is increased in the WT group after fear extinction, as compared to the group with the same virus before extinction, indicating increased synaptic strength. These data match the EM results (**Figure 5,** increase in total PSD area and volume in WT mice), and indicate that synaptic transmission can be effectively enhanced with PSD-95 over-expression *in vivo*, possibly supporting long-term fear extinction memory. This synaptic processes is impaired in S73A mutant group, likely explaining fear extinction deficits.

Furthermore, as we discussed in the introduction, it is possible that LTP in dCA1 is not necessary for long-term fear memory formation [[1–5]](https://www.zotero.org/google-docs/?uvhGq5). This is also supported by the observation that autophosphorylation-deficient CaMKII mutant mice (T286A mutants), which have severely impaired NMDAR-LTP in CA1 [[6]](https://www.zotero.org/google-docs/?8FbtKy), can form long-term memory after intensive training [[7]](https://www.zotero.org/google-docs/?cRr7mC) and **Figure 7**.

Finally, the data presented in the current manuscript does not support the idea that LTD in dCA1 enhances fear memory extinction. We did not observe any form of global synaptic weakening (structural or functional) after fear extinction. We found, however, bidirectional plasticity characterized by elimination of dendritic spines and growth of the remaining synapses (**Figure 2&5**). This form of plasticity is impaired in S73A animals.

*2. In figures 1, 2, 3 and 6 PSD-95 immunofluorescence is used to quantify PSD-95 in the dorsal CA1. From these measurements, several metrics are extracted (PSD-95+ density; total PSD-95; PSD-95+, PSD-95+ puncta...) and they slightly differ in the different figures. Some of these are easy to understand but others would benefit from a more detailed description. Please use consistent metrics and/or provide a rationale for using each of the different analysis methods.*

**ANSWER**: The metrics for PSD-95 levels have been unified. We show total PSD-95 (mean gray value of the microphotographs) for all experiments; as well as PSD-95^+^ puncta density (per μm of a dendrite) and intensity (per dendritic spine) in Thy1-GFP mice (**Figure 1**).

Ln. 495: “Custom-written Python scripts were used to analyze total PSD-95 (mean gray value of the microphotographs: all experiments); PSD-95+ puncta density (per μm of a dendrite) and mean gray value per dendritic spine in Thy1-GFP(M) (**Figure 1**).”

3. *In Figure 6, immunostaining with the antibody specific for PSD-95 S73 needs to be done in order to link CaMKII to this story.*

**ANSWER**: As advised by the reviewer, we performed the P-S73-PSD-95 immunostaining using the brain sections of the WT and T286A mice. The staining was performed on the brain sections of naive mice as we do not currently have sections from the trained T286A animals, and the T286A mouse line. Unfortunately, the results do not show lower P-S73-PSD-95 levels in the mutants, as compared to WT naive mice. This is likely due to low P-S73-PSD-95 levels in naive animals. Hence, we are not able to provide the data required by the reviewer. The inclusion of the figure 6 (now 7) is justified, however, by the published data showing that PSD-95(S73) is phosphorylated by the calcium and calmodulin-dependent kinase II (CaMKII) and the PSD-95(S73) phosphorylation requires αCaMKII autophosphorylation [(Gardoni et al., 2006; Steiner et al., 2008)](https://www.zotero.org/google-docs/?qn8vO3).

Although, in the revised manuscript this figure is still included, as it supports the whole story in our opinion, we are willing to exclude it if this is advised by the reviewer and editor.

4. *Line 220: No differences were observed in PSD-95 levels between mice with WT PSD-95 expression and PSD-95 (S73A). What about differences in PSD-95 levels in mice without viral injection, ie what is the level of overexpression?*

**ANSWER**: We apologize for this mistake in description. We did observe around 40% of overexpression of PSD-95 in WT and S73A groups as compared to the Control. This data is presented in **Supplementary Figure 2A-C**. In the revised version this is clearly stated in the results section with the reference to the Supplementary Figure 2.

p. 9: “We observed a significant increase in total PSD-95 protein levels in WT and S73A mice sacrificed before the fear extinction session as compared to the Control group sacrificed at the same time point (**Supplementary Figure 2A-C**).”

*5. Line 270: 'The S73A mutation impaired fear extinction-induced downregulation of dendritic spine density as well as dendritic spine and PSD growth'*

*What about the effect of the S73A mutation on the same metrics (Dendritic spine volume, PSD area and PSD volume)? It looks like the 5US groups are different.*

*Also, in S73A mice, the PSD area does significantly increase, which is contrary to the statement above; please explain.*

**ANSWER**: We found no significant differences between the 5US WT and S73A groups in dendritic spines parameters (**Supplementary Figure 2D-G**).

PDS surface area is indeed larger in S37A mice after extinction, however, not the volume of the dendritic spines and PSDs. This statement is corrected to precisely describe our observations.

Ln. 225: “We also found no significant changes of median dendritic spine volumes and PSD volumes (Figure 5D&F), and only a minor increase in the median PSD surface area in the S73A Ext group as compared to the S73A 5US animals (Figure 5E).”

6. *In the example picture shown in Figure 1 DEF, spine density and amount of PSD-95 puncta are visibly much lower in the stLM of conditioned animals (5US), this is not what is shown in the quantification at all (Figure 1GHIJ). Please provide an explanation for this and a representative example picture. Also, it would be good to show another example in a supplementary figure.*

**ANSWER**: In the revised manuscript we submit pictures of the stLM which better represent the quantification of dendritic spines and PSD-95 in these animals. The density of spines and total PSD-95 levels in stLM between 5US and Ext does not differ.

*Minor points:*

*7. Figure 4D: Impossible to see what is happening here; please present less (3-5) isolated examples of dendritic spines for each condition.*

**ANSWER**: We added reconstructions of isolated dendritic spines (**Figure 5C**).

*8. In the introduction, it is stated at line 75 that: ' Phosphorylation of PSD-95(S73) enables PSD-95 dissociation from the complex with GluN2B'. Another study found that PSD-95-S73A expression blocked the reduction in the NMDAR/PSD-95 interaction during chemical LTP in a manner that is dependent on CaMKII and calpain (Dore et al Plos One, 2014). This is consistent with the current study and the Steiner et al 2008 paper as well and should thus be mentioned and included in the citations.*

**ANSWER**: We would like to thank the reviewer for reminding us of this important study in line with the current results. We included it in the introduction.

Ln. 65. “Expression of phosphorylation-deficient PSD-95, with Serine 73 mutated to Alanine [PSD-95(S73A)], blocks the reduction in the NMDAR/PSD-95 interaction during chemical LTP in a manner that is dependent on CaMKII and calpain (Doré et al., 2014).”

9. *Line 268: 'synaptic changes observed in the WT mice resembled the changes found in Thy1-GFP(M) animals after contextual fear extinction'. Please be more specific, similarities were found in stratum oriens for the Thy1-GFP animals, which is where the SBEM experiments were done for Fig.4. What metrics exactly are similar?*

**ANSWER**: This is indeed an imprecise statement and is corrected now. We will indicate that the analysis of dendritic spines by confocal microscopy in Thy1-GFP and EM in WT mice observed decreased density of dendritic spines and increased volume of the remaining dendritic spines in stOri after extinction.

*Figure 2A: What is the signification of the H1, H2, H3 samples? Are these different mice? Why is there no band in the H2 sample?*

**ANSWER**: This information is added. These are samples from the animals sacrificed during (Ext15’) or before (5US) fear extinction, or a naive animal (N). There are pPSD-95 bands in all lines, just the band in the middle line is very weak.

*Line 65: PSD-95 is a major scaffolding protein at glutamatergic synapses*

**ANSWER**: This is corrected.

*Line 106: formation of fear extinction memory => extinction of fear memory*

**ANSWER**: This is corrected.

*Line 109: assess*

**ANSWER**: This is corrected.

*Line 211: 30-minute*

**ANSWER**: This is corrected.

*Line 460: co-localizes*

**ANSWER**: This is corrected.

*Reviewer #1: Yes: Kim Dore (Originally Reviewer 2 at Review Commons)*

*Reviewer #1: I reviewed this manuscript for Review Commons (I'm reviewer #2).*

*The authors responded to my comments in the response letter by saying that they will do all the requested changes and one other experiment I requested (regarding Fig. 6, third comment). If the authors are indeed making all the requested changes, I think this publication would be of interest to PLOS biology readers.*

**ANSWER**: As we mentioned above, we performed the P-S73-PSD-95 immunostaining using the brain sections of the WT and T286A mice. The staining was performed on the brain sections of naive mice as we do not currently have sections from the trained T286A animals, and the T286A mouse line. Unfortunately, the results do not show lower P-S73-PSD-95 levels in the mutants, as compared to WT naive mice. This is likely due to low P-S73-PSD-95 levels in naive animals. Hence, we are not able to provide the data required by the reviewer. The inclusion of the figure 6 (now 7) is justified, however, by the published data showing that PSD-95(S73) is phosphorylated by the calcium and calmodulin-dependent kinase II (CaMKII) and the PSD-95(S73) phosphorylation requires αCaMKII autophosphorylation [(Gardoni et al., 2006; Steiner et al., 2008)](https://www.zotero.org/google-docs/?VTyKVO).

Although, in the revised manuscript this figure is still included, as it supports the whole story in our opinion, we are willing to exclude it if this is advised by the reviewer and editor.

*One thing I'm unsure about is the answer to the first 'major point' I noted. I'm still very concerned about the effects of WT-PSD-95 expression on learning and memory as it's been shown in vitro that it blocks LTP and enhance LTD.*

*Even if overpexpression leads to 'only' a 40% increase in PSD-95 in vivo, I would expect it to have an impact on synaptic plasticity. A great way to test this would be to do electrophysiology on acute slices made from control or injected animals.*

*If LTP can be induced in PSD-95 injected animals, that would support the results presented and confirm that memory formation is intact in these animals.*

*I look forward to see how the authors will address all the comments in the revised manuscript.*

**ANSWER**: As we mentioned above, in the revised manuscript we added an experiment testing synaptic transmission in stOri before and after fear extinction in S73A and WT groups (**Supplementary Figure 3**). Our data show that fEPSP is increased in the WT group after fear extinction, as compared to the group with the same virus before extinction, indicating increased synaptic strength. These data match the EM results (**Figure 5,** increase in total PSD area and volume in WT mice), and indicate that synaptic transmission can be effectively enhanced with PSD-95 over-expression *in vivo*.

We also extensively discussed the consequences of PSD-95 overexpression in dCA1 *in vivo*. Indeed, this is truly fascinating why none of the tested cognitive processes are affected by PSD-95(WT).

Ln. 302: “To study the role of this plasticity in fear memory extinction we used dCA1-targeted overexpression of PSD-95(WT) and PSD-95(S73A). Interestingly, although PSD-95(WT) overexpression reduced dendritic spine density and increased median spine and PSD volume before fear extinction, the extinction-induced synaptic processes were preserved in PSD-95(WT) mice and fear extinction resulted in elimination of dendritic spines, as well as global increase of dendritic spine volume, PSD size and strengthening of synaptic transmission. Hence our research contradicts the earlier *in vitro* studies showing that PSD-95 over-expression results not only in synaptic growth, but also increased density of dendritic spines and impaired LTP [(El-Husseini et al., 2000; Stein et al., 2003)](https://www.zotero.org/google-docs/?XnyCWS). These differences between our study and *in vitro* experiments may result from the spatial constraints that are imposed on the neurons in the brain, but not *in vitro*, as well as the fact that neurons *in vivo* co-exist in complex networks that may induce homeostatic/compensatory mechanisms. Still, taking into account significant changes of dendritic spine morphology induced by PSD-95 overexpression *in vivo*, it is likely that computational power of such neurons is affected [(Bartol et al., 2015)](https://www.zotero.org/google-docs/?oIXtAA). As we found no effect of PSD-95(WT) overexpression on fear memory formation, recall, extinction or generalization; future studies are needed to discover whether any cognitive processes are affected by this presumed limitation of computational properties of the dCA1 neurons*.* It is also possible that our study further supports the notion that synaptic plasticity in dCA1 is not required for the formation of long-term memory [(Bannerman et al., 2014; Cały et al., 2021)](https://www.zotero.org/google-docs/?gLR7tk). “

[1.	Bannerman DM, Bus T, Taylor A, Sanderson DJ, Schwarz I, Jensen V, et al. Dissecting spatial knowledge from spatial choice by hippocampal NMDA receptor deletion. Nat Neurosci. 2012;15: 1153–1159. doi:10.1038/nn.3166](https://www.zotero.org/google-docs/?63mGLY)

[2.	Bannerman DM, Sprengel R, Sanderson DJ, McHugh SB, Rawlins JNP, Monyer H, et al. Hippocampal synaptic plasticity, spatial memory and anxiety. Nat Rev Neurosci. 2014;15: 181–192. doi:10.1038/nrn3677](https://www.zotero.org/google-docs/?63mGLY)

[3.	Hirsch SJ, Regmi NL, Birnbaum SG, Greene RW. CA1-specific deletion of NMDA receptors induces abnormal renewal of a learned fear response. Hippocampus. 2015;25: 1374–1379. doi:10.1002/hipo.22457](https://www.zotero.org/google-docs/?63mGLY)

[4.	Cały A, Śliwińska MA, Ziółkowska M, Łukasiewicz K, Pagano R, Dzik JM, et al. PSD-95 in CA1 area regulates spatial choice depending on age. J Neurosci. 2021 [cited 26 Jan 2021]. doi:10.1523/JNEUROSCI.1996-20.2020](https://www.zotero.org/google-docs/?63mGLY)

[5.	Kaganovsky K, Plitt MH, Yang R, Sando R, Giocomo LM, Ding JB, et al. Dissociating encoding of memory and salience by manipulating long-term synaptic potentiation. bioRxiv; 2022. p. 2022.01.04.474865. doi:10.1101/2022.01.04.474865](https://www.zotero.org/google-docs/?63mGLY)

[6.	Giese KP, Fedorov NB, Filipkowski RK, Silva AJ. Autophosphorylation at Thr286 of the alpha calcium-calmodulin kinase II in LTP and learning. Science. 1998;279: 870–873. doi:10.1126/science.279.5352.870](https://www.zotero.org/google-docs/?63mGLY)

[7.	Irvine EE, Vernon J, Giese KP. αCaMKII autophosphorylation contributes to rapid learning but is not necessary for memory. Nat Neurosci. 2005;8: 411–412. doi:10.1038/nn1431](https://www.zotero.org/google-docs/?63mGLY)
