# Supplementary material for: Phosphorylation of PSD-95 at serine 73 in dCA1 is required for extinction of contextual fear
Source: PLoS Biol. 2023 May 8;21(5):e3002106. doi: 10.1371/journal.pbio.3002106 (PMC10194913; doi:10.1371/journal.pbio.3002106)
Supplement: Attachment — Submitted filename: PLOS Biol_rebuttal_final.docx [file pbio.3002106.s009.docx]

1) TITLE: We think that 'Serine' should not be capitalized in the title. If you agree, we suggest you change the title to:  "Phosphorylation of PSD-95 at serine 73 in dCA1 is required for extinction of contextual fear"

**ANSWER**: The title was corrected as advised.

2) FINANCIAL DISCLOSURES: Please update your financial disclosures statement to describe the role of any sponsors or funders in the study design, data collection and analysis, decision to publish, or preparation of the manuscript. If the funders had no role in any of the above, include this sentence at the end of your statement: "The funders had no role in study design, data collection and analysis, decision to publish, or preparation of the manuscript
**ANSWER**: This statement was added.

3) DATA REQUEST: Thank you for including the raw western blot images related to your manuscript. Can you please annotate this data, to clearly indicate which figure panel the image corresponds to, to note the loading order, antibody used, and molecular weights?  Please add these annotations without obscuring the lanes. Please carefully read our guidelines for how to prepare and upload this data: <https://journals.plos.org/plosbiology/s/figures#loc-blot-and-gel-reporting-requirements>
**ANSWER**: The raw picture of western blot was annotated as advised. Please, see OSF (<https://osf.io/cgfa9/>).

4) DATA REQUEST: Thank you also for uploading the raw data underlying your figures on OSF. Can you please reference this dataset in each figure legend? For example, to each figure legend (including supplemental figure legends), you can add the sentence "the data underlying this figure is available from OSF (<https://osf.io/cgfa9/>)
**ANSWER**: This information is added to each figure legend.

5) ABSTRACT: Please note that per journal policy, the model system/species studied should be clearly stated in the abstract of your manuscript.  Please update it accordingly.

**ANSWER**: The abstract was corrected as advised.
